# Determining regional brain growth in premature and mature infants in relation to age at MRI using deep neural networks

**DOI:** 10.1038/s41598-023-40244-z

**Published:** 2023-08-15

**Authors:** Farzad Beizaee, Michele Bona, Christian Desrosiers, Jose Dolz, Gregory Lodygensky

**Affiliations:** 1 Software and IT Department, École de Technologie Supérieure, Montreal, QC H3C 1K3 Canada; 2 Department of Pediatrics, CHU Sainte-Justine, University of Montreal, Montreal, QC H3T 1C5 Canada; 3 Canadian Neonatal Brain Platform, Montreal, QC Canada

**Keywords:** Computer science, Paediatric research

## Abstract

Neonatal MRIs are used increasingly in preterm infants. However, it is not always feasible to analyze this data. Having a tool that assesses brain maturation during this period of extraordinary changes would be immensely helpful. Approaches based on deep learning approaches could solve this task since, once properly trained and validated, they can be used in practically any system and provide holistic quantitative information in a matter of minutes. However, one major deterrent for radiologists is that these tools are not easily interpretable. Indeed, it is important that structures driving the results be detailed and survive comparison to the available literature. To solve these challenges, we propose an interpretable pipeline based on deep learning to predict postmenstrual age at scan, a key measure for assessing neonatal brain development. For this purpose, we train a state-of-the-art deep neural network to segment the brain into 87 different regions using normal preterm and term infants from the dHCP study. We then extract informative features for brain age estimation using the segmented MRIs and predict the brain age at scan with a regression model. The proposed framework achieves a mean absolute error of 0.46 weeks to predict postmenstrual age at scan. While our model is based solely on structural T2-weighted images, the results are superior to recent, arguably more complex approaches. Furthermore, based on the extracted knowledge from the trained models, we found that frontal and parietal lobes are among the most important structures for neonatal brain age estimation.

## Introduction

MRI is increasingly used in neonates as it provides a wealth of information vastly superior to ultrasound and CT scans. Simple preparation with sedation achieved by milk alone and swaddling is sufficient to guarantee high-quality, motionless imaging data without needing any anesthetics^1^. However, it can be challenging to analyze neonatal brain MRIs due to the lack of readily-available and age-specific references. Having a fast and simple tool that assesses brain maturation during such a period of extraordinary changes would be immensely helpful. Brain segmentation using standard image analysis tools has been tremendously useful in analyzing brain development in the last few decades. Already in 1998, Huppi et al.^2^ used the k-nearest-neighbor (k-NN) classification to show how gray matter volumes correlated significantly with postmenstrual age at MRI, more so than unmyelinated white matter.

Since then, more advanced analyses studying cortical folding in preterm infants have shown how this process is tightly controlled during the last trimester with a strong correlation to postmenstrual age. As described by Dubois et al.^3^, the general proportion of sulci compared to the brain size was found to correlate to the postmenstrual age of the infant. Shimony et al.^4^ also demonstrated that the general curvature and sulcal depth of the brain were highly correlated with age. More recently, Galdi et al.^5^ segmented the brain into 81 regions and then extracted related features from structural MRI and diffusion MRI, which served to predict postmenstrual age (PMA) at scan based on inter-regional similarities. The advantage of these techniques is that each step can be visualized and validated. On the other hand, their use in daily clinical practice is not viable due to the many processing steps and high computing time.

In recent years, deep learning-based models have demonstrated an astonishing performance on a wide range of medical problems such as classification, detection, and segmentation^6,7^. Indeed, tools based on deep learning are readily available for both research and daily clinical practice. Once properly trained and validated, they can be installed on practically any computer and provide holistic quantitative information in a matter of minutes. Furthermore, having a learning methodology based solely on structural T2-weighted images would guarantee its generalizability, as any center with access to an MRI machine would be able to secure data with sufficient quality for interpretation. Widely-used in other medical research fields, deep neural networks (DNN) have also found their way to the task of brain estimation using medical images. Recently, several brain age estimation approaches based on deep learning were proposed for brain age estimation in fetuses, infants, and adults. Peng et al.^8^ proposed to use a simple fully convolutional network for this task, arguing that it does not require a very deep neural network. Furthermore, they advocated that smaller networks would be more robust for small datasets. In their study, Cheng et al.^9^ leveraged a ranking loss term and a two-stage cascaded 3D convolutional neural network (CNN) to improve the accuracy of brain age estimation. A transformer-shaped network was proposed by He et al.^10^ to fuse local features from smaller patches with global features by using an attention mechanism. Shi et al.^11^ employed an attention-based deep residual network with structural MRI to predict the brain age. They also computed the predictive uncertainty using an ensembling strategy to estimate the model’s confidence as a marker for fetal brain anomaly detection. Hong et al.^12^ employed multiplanar slices in orthogonal directions and a test-time-augmentation technique to predict the brain age based on each slide. The most frequent value among the predictions was used as the estimated age. More recently, Taoudi-Benchekroun et al.^13^ exploited Diffusion and T2-weighted MRI images to generate individual brain connectivity maps, which were later used by a deep network to predict age.

Despite the growing interest in brain age estimation using DNNs, yet due to the immense power of these networks to handle a vast quantity of data, they tend to resort to multimodal data, which is not always accessible, or too complex deep neural networks that might limit their reproducibility and highly increase their computation resources. Another major deterrent of DNN for radiologists is their lack of interpretability. Indeed, it is vital that the structures driving the results are detailed and compared with available literature. Furthermore, identifying important features is of paramount importance in case of brain injury, where regional changes could greatly affect the accuracy of results. Unfortunately, existing approaches based on deep models tend not to provide a mechanism to identify the most important structures for the age estimation task.

**Figure 1:**
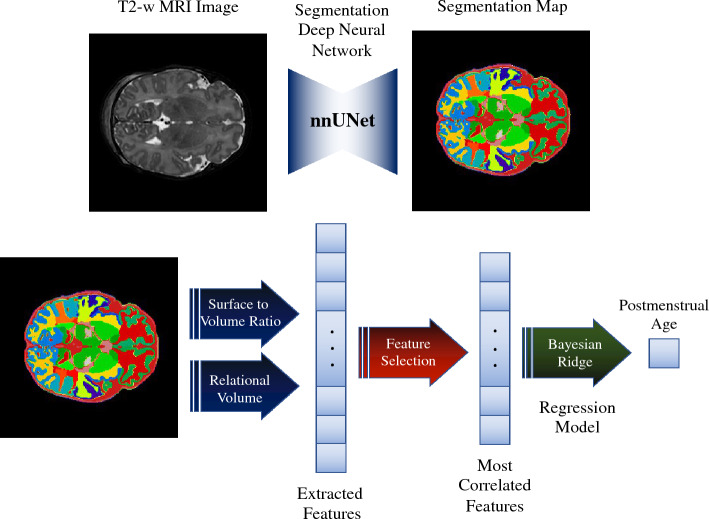
Overview of the proposed method. First, we employ 3D T2 weighted MRI as input for the segmentation model. The generated segmentations were then used to extract the volume and surface of each class (i.e., 87 classes representing 87 brain regions). Using these measures, we calculated the proposed metrics, i.e., the surface to volume ratio and the relational volume. After this step, we resorted to a feature selection strategy, known as PFI, to keep the most important regions for brain age estimation. Finally, we used a Bayesian ridge regression model to get the predicted postmenstrual age.

Motivated by the aforementioned limitations, we designed a learning-based pipeline to predict neonatal brain age using T2-weighted MRIs. The proposed approach first segments the T2-weighted MRIs into 87 cortical and sub-cortical structures, which are then used to extract features associated with volume and gyrification. These features, termed relational volume and surface to volume ratio, are informative for brain age estimation yet are easy to calculate. Finally, a machine learning regression model can be trained to predict brain age using the extracted features from the segmented MRIs. The overview of the proposed pipeline is shown in Fig. 1. The proposed pipeline predicts brain age in a fast and accurate manner. It is also interpretable, as we can identify the most important features driving the results using popular features selection techniques, such as Permutation Feature Importance (PFI)^14^. It is important to note that the term ”interpretability” in our context refers to the ability to extract and understand the significant factors and features utilized by the model in predicting brain age using MRI data. This extracted information holds the potential for examination and validation through surveys conducted with end-users, such as radiologists. Additionally, it can serve as a valuable resource in guiding future research focused on brain development during the last trimester or identifying landmarks for neonatal brain age.

## Results

### Main results

We used the developing Human Connectome Project (dHCP)^15^ dataset to empirically validate the proposed method. Following standard practices in machine learning, the data was split into three distinct random subsets, resulting in independent train, validation and test sets. The train data, which includes 60% of the scans (334 images), was first used to train the segmentation and regression models for brain age estimation. Then, the validation data, which includes 15% of the data (84 images), was employed to find the optimal parameters and select the best models. Last, the remaining 25% of the data (140 images) was used for testing the method and comparing to other approaches.

The quantitative results achieved by the proposed framework for brain age estimation are reported in Table 1. Recent works that solely employ structural MRI for the same task are also included for comparison purposes. From these results, we observe that the proposed approach largely outperforms recent literature in terms of the Mean Absolute Error (MAE), achieving a value of 0.46 weeks on the independent test set. Compared to relevant works (2DTTA, ARN, and GLT), our approach brings between 5 and 11% improvement. A noteworthy point to highlight is the fact that existing approaches predict brain age directly from the structural MRI, which makes the interpretation of the predictions difficult. In contrast, as our method employs the segmentation results to derive two per-region features, these can be selected according to their correlation with brain and gestational age. As we show later in our empirical validation, this allows shedding light on the regions that have a significant impact on brain age estimation.

**Table 1:** Quantitative results compared to state-of-the-art learning-based brain age estimation methods.

Model	MAE	R^2^	RMSE
2D Test-time-Augmentation (2DTTA)^12^	0.52 ± 0.40	0.96	0.66
Attention-based residual network (ARN)^11^	0.57 ± 0.44	0.95	0.75
Global–local transformer (GLT)^10^	0.51 ± 0.39	0.96	0.64
Proposed method	**0.46** $\pm$ **0.37**	**0.97**	**0.60**

**Figure 2:**
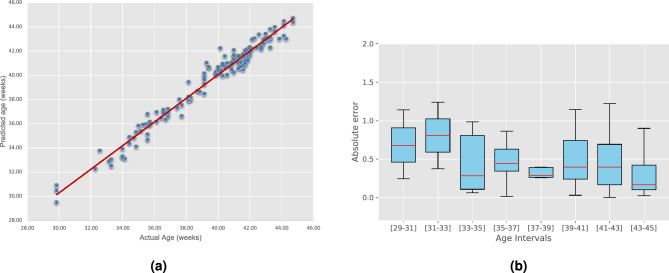
(**a**) predicted age versus real age; (**b**) prediction error for different age intervals.

Figure 2 depicts additional results obtained by our method. Figure 2a shows the relation between the predicted versus the real age, which shows a high correlation between both. Indeed, these visual results are supported by the high determination score ($R^2$) of 0.97 obtained by the proposed approach. Despite differences in the mean absolute error across age intervals (Fig. 2b), we can see that these are typically consistent, with slightly lower means as the age increases. Last, we observe that the MAE obtained by our approach for the “male” and “female” populations are quite similar (0.46 and 0.47 weeks, respectively), indicating that our method is gender agnostic.

### On the importance of different regions

**Figure 3:**
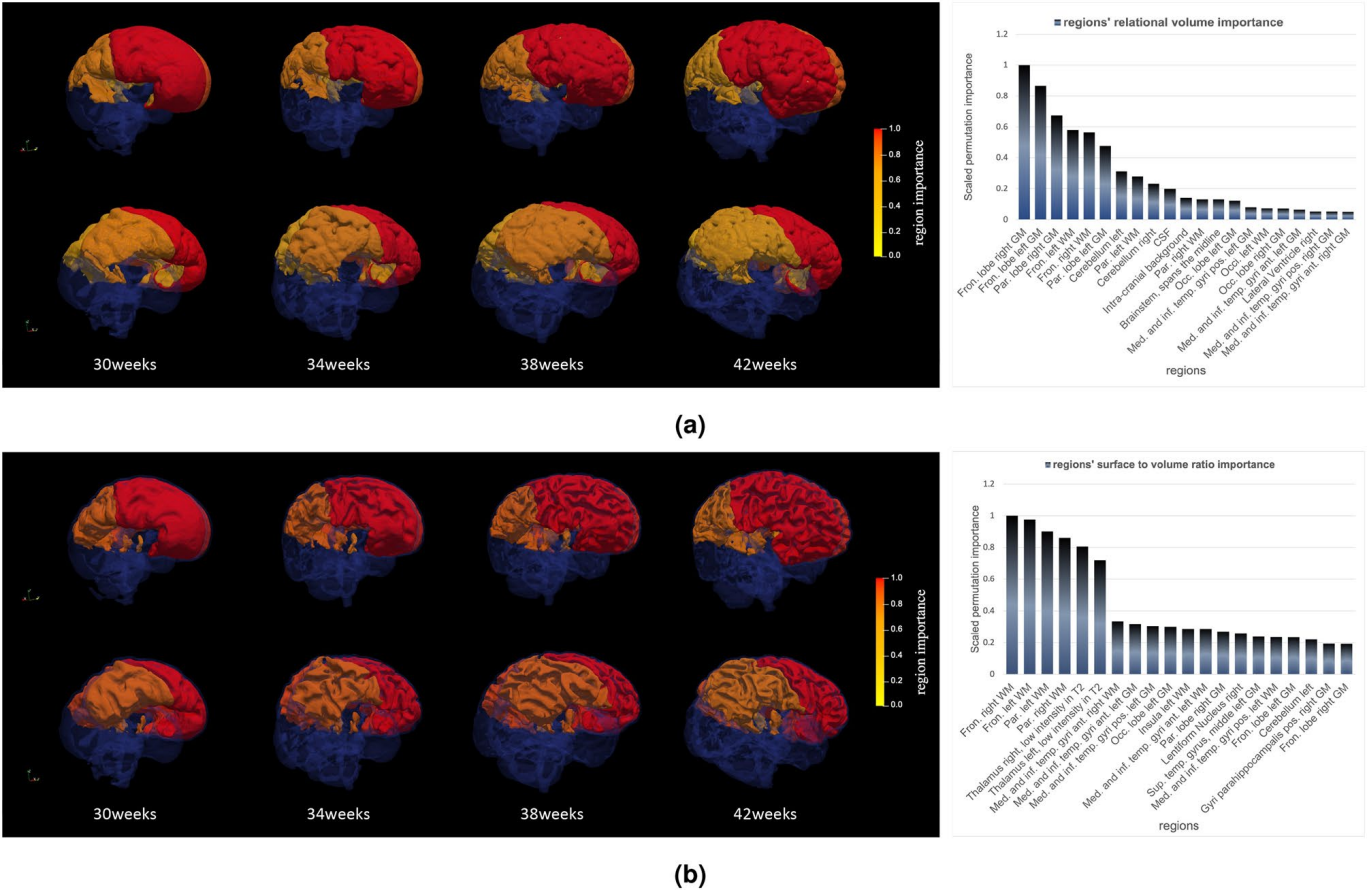
Histogram showing the brain regions with the best scaled PFI (on the right) and a 3D representation of these regions colored by their PFI value (on the left). The brains are selected from the dHCP dataset aged (from left to right) 30, 34, 38, and 42 weeks. The first row (**a**) shows that the most significant brain structures for RV were the frontal lobe right and left gray matter (Right PFI 0.1, Left PFI: 0.86), the parietal lobe right gray matter (PFI: 0.67), the frontal lobe left and right white matter (Left PFI: 0.58, Right PFI 0.56) then the parietal lobe left gray matter (PFI: 0.48). The second row (**b**) shows that the most significant brain structures for SVR were the frontal lobe right and left white matter (Right PFI 1.0, Left PFI: 0.97), the parietal lobe right white matter (PFI: 0.9), then the right and left thalamus (Right PFI 0.8, Left PFI: 0.71).

One of the advantages of the proposed method is that it is interpretable in terms of the importance of each region and its corresponding features. therefore, it is possible to extract information from the trained regression model, which provides a possible landmark to look for predicting brain age. To understand the importance of each region-feature pair in brain age estimation, two regression models are trained with the surface to volume ratio (SVR) and relational volume (RV) separately. Then, the importance of each region for each of the features is calculated using a permutation feature importance approach. Finally, the importance of each region is re-scaled to be constrained between 0 and 1, for better interpretability. These observations can then be used as a valid biomarker for neonatal brain age estimation. The most important regions and their importance for relational volume and surface to volume ratio are shown in Fig. 3. Based on the obtained regions’ importance, the extracted relational volumes (RV) of frontal lobe gray and white matter and parietal gray matter have the most correlation with neonatal brain age. The scaled importance factors for these structures are higher than 0.4, and the remaining structures have lower scaled importance. Additionally, we can also observe that frontal and parietal lobe white matter and thalamus surface to volume ratios (SVR) are the most informative regions for predicting brain age, with scaled importance factors of more than 0.6.

### Comparison to existing brain age bio-markers

In this section, we compared the two proposed features, i.e., Relational Volume (RV) and Surface to Volume Ratio (SVR) to existing biomarkers commonly used to estimate brain age: sulcal depth, cortex thickness, and curvature. These experiments, whose results are reported in Table 2, demonstrate that the proposed features indeed outperform other proposed metrics in the literature. In particular, compared to the cortex thickness and curvature, the improvements brought by the proposed features are substantial.

**Table 2:** Comparison of the quantitative performance obtained by the proposed features and existing features for brain age estimation.

	MAE	$R^{2}$	RMSE
Sulcal depth	0.67 $\pm$ 0.52	0.93	0.85
Cortex thickness	1.37 $\pm$ 1.08	0.72	1.75
Curvature	1.02 $\pm$ 0.76	0.85	1.27
RV and SVR (Proposed)	**0.50** $\pm$ **0.38**	**0.96**	**0.62**

### Performance on low labeled data regime

It is well known that deep learning models require large amounts of training labeled data to work satisfactorily. Nevertheless, due to several factors, including time-consuming processes and annotator variability, having access to such large curated datasets is not always easy. Thus, we believe that it is important to investigate the effect of low labeled data regime on the performance of different approaches. To this end, we repeated the training of several approaches under several amounts of labeled training data, and reported their results in Table 3. An interesting observation is that, particularly in the most extreme scenario (i.e., only 10 labeled scans were used for training), the performance gap between the proposed method and existing literature is substantially large. More concretely, recent works achieve an MAE ranging from 1.37 to 2.39 weeks, whereas our method can predict the brain age with an MAE of 0.74 weeks, which represents less than half of the value obtained by compared approaches. We also observed that even though the difference between the different approaches is reduced as the number of labeled samples increases.

**Table 3:** Quantitative performance of different brain age estimation methods based on a fraction of labeled data.

$\#$ Training images	Method	DSC	MAE	$R^{2}$	RMSE
10 scans	2DTTA^12^	–	1.37 ± 1.21	0.70	1.82
	ARN^11^	–	2.39 ± 1.52	0.14	2.7
	GLT^10^	–	1.77 ± 1.30	0.57	2.2
	Proposed	0.846	**0.74** $\pm$ **0.57**	**0.92**	**0.94**
20 scans	2DTTA^12^	-	0.84 ± 0.78	0.88	1.15
	ARN^11^	–	1.39 ± 1.13	0.70	1.57
	GLT^10^	–	1.09 ± 0.80	0.83	1.36
	Proposed	0.875	**0.68** $\pm$ **0.54**	**0.93**	**0.86**
40 scans	2DTTA^12^	-	0.70 ± 0.64	0.92	0.95
	ARN^11^	–	0.96 ± 0.83	0.86	1.14
	GLT^10^	–	0.80 ± 0.68	0.90	1.05
	Proposed	0.895	**0.57** $\pm$ **0.45**	**0.95**	**0.73**
60 scans	2DTTA^12^	–	0.61 ± 0.50	0.95	0.79
	ARN^11^	–	0.96 ± 0.79	0.87	1.11
	GLT^10^	–	0.73 ± 0.54	0.93	0.91
	Proposed	0.901	**0.56** $\pm$ **0.40**	**0.95**	**0.68**
100 scans	2DTTA^12^	–	0.60 ± 0.47	0.95	0.76
	ARN^11^	–	0.76 ± 0.59	0.92	0.93
	GLT^10^	–	0.58 ± 0.44	0.95	0.73
	Proposed	0.909	**0.51** $\pm$ **0.39**	**0.96**	**0.64**

We repeated the training of the segmentation and regression models with only a fraction of data to analyze the amount of data required for training and its effects on segmentation and brain age estimation. It is important to investigate the effect of a low data regime since medical data is limited, costly, and hard to obtain. Also, the performance of the model based on the low data shows its generalizability and robustness, which is a crucial factor for medical image analysis. For the low data regime, each time, 10, 20, 40, 60, and 100 scans were selected from the training split to train the models respectively. Also, 40% of the training data was used as the validation set. However, the models were tested with the whole test split (140 scans). Besides, we preserved the same images for training and validation sets of different experiments for a fair comparison. We also tried this configuration for all comparing methods for better analysis.

### The impact of different backbones

The choice of different segmentation or regression models can arguably have a significant impact on the final performance. To assess these potential performance differences, we investigate several segmentation and regression models in our framework, whose results are reported in Table 4.

**Table 4:** Results using different segmentation and regression backbones.

	Without feature-selection			With feature-selection		
	MAE	$R^{2}$	RMSE	MAE	$R^{2}$	RMSE
Segmentation models						
DRAW-EM^16^	0.51 $\pm$ 0.40	0.96	0.65	0.50 $\pm$ 0.39	0.96	0.63
UNet^18^	0.52 $\pm$ 0.40	0.96	0.65	0.50 $\pm$ 0.40	0.96	0.63
nnUNet^19^	**0.50**	**0.96**	**0.62**	**0.46**	**0.97**	**0.60**
Regression models						
Kernel Ridge^20^	0.61 $\pm$ 0.50	0.94	0.79	0.56 $\pm$ 0.44	0.95	0.72
ElasticNet^21^	0.82 $\pm$ 0.55	0.91	0.98	0.82 $\pm$ 0.55	0.91	0.98
GradientBoosting^22^	0.57 $\pm$ 0.48	0.95	0.73	0.58 $\pm$ 0.49	0.95	0.76
SVM Regressor^23^	0.75 $\pm$ 1.00	0.86	1.25	0.61 $\pm$ 0.81	0.91	1.01
MLP Regressor^24^	0.58 $\pm$ 0.41	0.95	0.76	0.55 $\pm$ 0.45	0.95	0.72
Bayesian Ridge^25^	**0.50** $\pm$ **0.38**	**0.96**	**0.62**	**0.46** $\pm$ **0.37**	**0.97**	**0.60**

In terms of the segmentation model, we first evaluated the performance of the brain labels extracted by DRAW-EM (Developing brain Region Annotation With Expectation-Maximization)^16,17^, a popular software for neonatal brain MR image segmentation. Furthermore, we included two well-known medical image segmentation networks (UNet^18^ and nnUNet^19^), which achieve state-of-the-art results in a broad span of medical segmentation problems. Note that both UNet and nnUNet were trained with the labels generated by DRAW-EM. From the results in Table 4,*top* we can observe that despite the segmentation results might be different across networks nnUNet achieved 0.920 of DSC versus 0.907 of UNet, the MAE values obtained without feature selection are almost identical. Nevertheless, when the most correlated features were selected, the performance differences between nnUNet and UNet are larger. This indicates that the feature selection process indeed plays an important role in removing noise from uncorrelated, or not as much correlated features. Furthermore, regardless of the segmentation method employed, the obtained results outperform the current literature, whose achieved MAE results were 0.51 (GLT), 0.52 (2DTTA), and 0.57 (ARN). An interesting and surprising observation is that, while UNet and nnUNet were trained from DRAW-EM segmentation labels, they typically yield better results. Thus, these results indicate that even though the brain age estimation accuracy increases with the segmentation accuracy, the proposed method does not require very complex segmentation networks to achieve satisfactory performances, which contrasts with prior works.

We now evaluate the effect of different regression models in Table 4, *bottom*. In contrast to the previous observations regarding the segmentation model, the choice of the regression approach significantly impacts brain age prediction accuracy. In particular, the differences between ElasticNet (worst) and Bayesian Ridge (best) as regression approaches are equal to 0.32 and 0.36 weeks without and with feature selection, respectively. This indicates that even though our pipeline is sensitive to the choice of the regression method, the achieved results by most models can be considered satisfactory and shows that our pipeline is model-agnostic. However, we suggest that a proper validation must be conducted to select the best strategy. Similar to the segmentation scenario, the step of feature selection further improves brain age prediction, which demonstrates its usefulness in finding correlated features and removing potential sources of noise during the learning process.

## Discussion

Relational volume (RV) and surface to volume ratio (SVR) in 87 cortical and sub-cortical brain classes were extracted using a fully automated pipeline built on a combination of machine learning approaches to determine postmenstrual age at MRI, which achieved an MAE of 0.46 weeks. For this purpose, the dHCP database^15^ has been used which includes 558 neonatal brain T2-weighted MRIs ranging from 29 to 45 weeks coupled with brain regions’ contours extracted from DrawEM^16^. First, using 60% and 15% of the scans as training set and validation set respectively, a segmentation network (nnUNet^19^) was trained to segment the brain MRIs into 87 different regions. Then using the segmented regions, RV and SVR features, which are easy to calculate, are obtained for each region and the ones with the highest correlation with brain age are selected to train a regression model. Once the model was trained, we evaluated the proposed pipeline for neonatal brain age estimation using the remaining 25% of scans. Finally, we wanted to assess critical structures and morphometric features indicative of brain age estimation, and using PFI we found that frontal and parietal lobes and thalami were among the most important structures driving these results in a cohort of healthy term and preterm infants.

Adding the proposed measurements brought a substantial improvement compared to more established biomarkers, such as cortex thickness or curvature. We also evaluated several regression networks and showed the advantage of the Bayesian Ridge approach. We believe that the benefit of using it may be linked to its potential to handle limited data^25^. Still, it is essential to note that the results remain very good for most of the regression models showing that the pipeline is not entirely dependent on a specific regression model (Table 4). Furthermore, we have assessed the effect of having different segmentation networks in the first stage of the proposed pipeline, and despite differences in the segmentation results, the choice of this backbone does not have a significant impact on the final brain age estimation task.

The use of permutation feature importance unveiled critical structures and morphometric features used to determine brain age between 29 and 45 weeks postmenstrual age at scan (Fig. 3). The most significant structures when using the relation volume (RV) feature were both frontal gray and white matter lobes, parietal gray matter lobes, parietal left white matter, and the cerebellum. These findings are well aligned with the study by Gui et al.^26^, showing how cortical gray matter and cerebellum using conventional metrics had the fastest growth structures during the period of prematurity. Furthermore, Hong et al.^12^ employed a saliency visualization method in fetal MRIs and also found that the cortex and ventricles were major regions for age estimation. Unfortunately, cortical parcellation was not available in both studies, so comparison with the frontal and parietal lobes is not possible.

The most significant morphometric features by quantifying the surface to volume ratio (SVR) were frontal and parietal white matter and both thalami (Fig. 3). The importance of thalamic maturation has already been described by Deprez et al.^27^ with a strong age estimation potential shown by root mean squared errors (RMSEs) of 1.41 weeks in newborns between 29 and 44 weeks. Interestingly, it is not frontal and parietal cortical but white matter folding that were the most significant morphometric features. This might be explained by cortical thickness in relation to image acquisition parameters with significant partial volume effects between gray matter and cerebro-spinal fluid. Indeed the boundary between gray matter and white matter was preferred in several prior works in this age range^28,29^. Similarly, using conventional image analysis tools major gyrification growth has been identified in the frontal lobe ($R^2$=0.84) with an even higher correlation in the Temporal-parietal-occipital region ($R^2$=0.9). Hill et al.^30^ described a similar major cortical expansion in frontal and parietal and temporal relative to others when comparing newborns to young adults.

Last, we would like to highlight that, for the sake of fairness, we have conducted experiments not only on the proposed approach but also on three recent relevant works. In particular, the four evaluated methods are trained and evaluated under the exact same conditions and with the same patients, which makes the results across models directly comparable. Under this scenario, the proposed pipeline outperformed recent methods, setting new state-of-the-art results for the task of image-based brain age estimation using only a single MRI modality. Last, it is noteworthy to mention that there exist other methods that were not included in the empirical validation, mainly due to different settings, e.g., multimodal images^5,13^. Nevertheless, the results obtained by these approaches were far from the performance achieved by our pipeline. For example, Liu et al.^31^ evaluated several structures and obtained a maximum MAE of 1.19 weeks using T1-weighted images alone. Galdi et al.^5^ obtained an MAE of 0.70 weeks in newborns scanned between 38 and 45 weeks postmenstrual age based on multimodal data from T1 and T2-weighted imaging and Multi-shell diffusion MRI using a linear regression model with elastic net regularization. Taoudi-Benchekroun et al.^13^ achieved similar results with an MAE of 0.72 weeks in newborns scanned from 37 to 45 weeks from the dHCP cohort combining multimodal data extracted from T2-weighted and Multi-shell diffusion MRI; While our method achieved a better MAE (0.46) despite of considering a wider age range.

### Limitations

This study characterized a healthy cohort of preterm and term infants. The results may be different in preterm infants with selective injury. The interesting aspect of PFI is that we now have identified critical structural and morphometric features in the neonatal brain driving brain age assessment. It will be critical in the future to assess the reliability of this approach in newborns with brain injury. When studying the impact of prematurity itself on cortical folding, the main structures affected were the insula, superior temporal sulcus, and ventral portions of the pre- and postcentral sulci, features that are very different from the ones we identified to determine brain age. It will be critical before translating these powerful tools into daily clinical practice to determine their efficacy in several clinical situations such as diffusion and cystic white matter injury and that interpretability be always accessible.

Compared to deep learning models trained end-to-end, our method considers feature learning and brain age estimation as two separate steps. A drawback of this strategy is that the learned features may not be optimal for the prediction task. However, it also brings the significant advantage of making the results of method more interpretable, since it enables identifying the regions and imaging features that are more important for brain age estimation.

In this study, we validated our method on the developing Human Connectome Project (dHCP) dataset. However, as it relies on structural characteristics (e.g., volume and gyrification) which are not dataset-dependant, our method could be easily adapted to any other dataset. Furthermore, with recent advancements in domain adaptation and harmonization, segmentation networks trained on given data can be easily adapted to MRIs from other datasets, making our brain age estimation method generalizable.

## Materials and methods

In this section we present the datasets used and detail the experiments and methodology employed in this work.

### Dataset

To empirically validate the proposed approach for brain age estimation we resorted to the T2-weighted (T2-w) images from the developing Human Connectome Project (dHCP)^15^ data. Imaging was carried out on 3T Philips Achieva using a dedicated neonatal imaging system. There are 558 sessions with T2-w images that passed QC from 505 different subjects. Infants from the DHCP database were recruited and imaged at the Evelina Neonatal Imaging Centre, London. Informed parental consent was obtained for imaging and data release, and the study was approved by the UK Health Research Authority^32^. The medical ethical review board of CHUSJ hospital approved the study. Also, all the methods were performed in accordance with the relevant guidelines and regulations.

The volumes were segmented into 87 regions using the atlas-based segmentation approach known as DRAW. To provide structural priors, manually labeled atlases with expert neuroanatomist annotations are registered to the volume. Afterward, segmentation is carried out using an Expectation-Maximization technique that combines the volume’s intensity model with structural priors^16,17^. Among those 505 subjects, 222 subjects are female and the remaining 283 are male. They are born between $24^{+2}$ weeks and $42^{+2}$ weeks of gestation, and scanned at age from $29^{+2}$ weeks to $45^{+1}$ weeks. The available dHCP dataset was split into three completely distinct sets of subjects for training, validation, and testing which include 334, 84, and 140 scans respectively.

### Methodology

Prediction of neonatal postmenstrual age at MRI using T2-weighted brain images consists of three parts. First, MRI images are segmented into different cortical and intra-cortical sub-structures using a state-of-the-art deep neural segmentation network. After that, features that are representative of neonatal brain age estimation are extracted for each structure separately. Once proper features for different substructures are extracted, a regression machine learning approach is used to predict the neonatal brain age. Each of these steps are detailed below.

#### Brain segmentation

Convolutional neural networks (CNN) have proven to be a powerful tool for image segmentation^33^. The use of CNNs can significantly improve the accuracy of brain segmentation which is a critical task in neuroimaging with various applications in both clinical and research settings^34,35^. CNNs can learn to automatically identify complex patterns and relationships within brain images, enabling precise and efficient segmentation of different brain structures. Motivated by recent advancements in deep learning segmentation models^18,19,36,37^, a CNN model was trained to segment the brain into 87 different regions by using the segmentation masks provided by DRAW-EM. The aim of the segmentation model is to assign a unique structure class to each voxel of the brain MRI so that there exists a maximum overlapping with respect to the ground-truth masks. This is achieved by optimizing the network parameters to minimize a segmentation loss function (e.g., Cross-Entropy or Dice loss^38^) for each pair of the input volume ${\varvec{{x}}}_n$ and its corresponding segmentation volume ${\varvec{{y}}}_n$ as:

1 $$\begin{aligned} \min _{\theta } {\frac{1}{N}}\sum _{n=1}^{N}{\mathcal {L}_{seg}\left( \ {\widetilde{\textbf{y}}}_n=f_{seg}\left( {\varvec{{x}}}_n|\theta \right) ,\ {\ {\varvec{{y}}}}_n\right) }. \end{aligned}$$

Where *N* is the number of training samples, $f_{seg}(.|\theta )$ is the segmentation network containing a set of learnable parameters $\theta$, $\mathcal {L}_{seg}$ is the segmentation loss function and ${\widetilde{\textbf{y}}}_n$ the predicted segmentation.

#### Extracting representative features

In the literature, different landmarks are reported to be important for neonatal brain age estimation. Nevertheless, they are either inaccurate or very hard and time-consuming to calculate^39^. In this work, we propose to use two different landmarks for segmented structures which are representative for brain age estimation.

The first type of features which are used to predict postmenstrual age from brain MRI is the relational volume (RV) of each structure compared to the whole brain. In particular, RV is computed by dividing the number of voxels assigned to a given structure by the total number of voxels contained in all the structures. The advantage of relational volume to absolute volume is that it removes the effect of head size for age prediction. Furthermore, it is very easy to compute, as basically involves summing the voxels across structures and a single division. As it can be seen in the top plot of Fig. 3, there is a high correlation between the relational volume of several structures in the brain and postmenstrual age. This indicates that this metric has the potential to be a good indicator of brain age.

Another feature that is reported in the literature as a good indicator for brain age estimation is how gyrified and folded the brain cortex and other structures are. For example, neonates’ brain cortex will have more gyrification as the preterm neonates grow. Nevertheless, computing the gyrification index and estimating the neonatal brain age based on this biomarker is very slow and hard to achieve^39^. In this work, we propose using a novel measurement that is highly correlated with gyrification, but it is much easier to calculate. The ratio of the surface of a structure to its volume shows how folded a structure is. Thus, the surface to volume ratio (SVR) of a folded structure would be higher than the surface to volume ratio of a smooth and unfolded structure. Besides, this metric can be calculated easily by simply counting the number of voxels on the surface of a structure (i.e., neighboring voxels labels are different) and dividing it by the corresponding structure volume. We refer to this structure feature as SVR. Similarly, the plot in the bottom right of Fig. 3 illustrates that there exists a strong correlation between the SVR of several regions and the postmenstrual age.

Based on these observations, the above-mentioned two features, i.e., relational volume (RV) and surface to volume ratio (SVR), were calculated for each of the 87 structures and they are later employed for training the brain age regression model.

#### Postmenstrual age regression

To predict the neonatal brain age based on the extracted features from previous steps, we used a machine learning regression approach known as Bayesian Ridge^25^. The ridge regression model is defined as:

2 $$\begin{aligned} {\arg \min }_{\omega } \Vert Z-\chi \omega \Vert _{2}^{2}+\lambda \Vert \omega \Vert _{2}^{2} \end{aligned}$$

where *Z* represents the postmenstrual ages, $\chi$ are the extracted features from previous steps, $\omega$ the model parameters, and $\lambda$ a balancing term, which imposes a penalty on the size of the coefficients which makes the regression model more robust and generalized. Bayesian Ridge regression models linear regression using probability distribution rather than point estimates, which allows it to handle limited or poorly distributed data. To do so, the output *y* is assumed to be Gaussian distributed around $\chi \omega$:

3 $$\begin{aligned} p(y \mid \chi , w, \alpha )=\mathcal {N}(y \mid \chi w, \alpha ) \end{aligned}$$

Also, Bayesian Ridge regression estimates a probabilistic model of the regression problem using spherical Gaussian prior for the coefficients.

4 $$\begin{aligned} p(w \mid \lambda )=\mathcal {N}\left( w \mid 0, \lambda ^{-1} \textbf{I}_{p}\right) \end{aligned}$$

where the priors over $\alpha$ and $\lambda$ are chosen to be gamma distributions.

To improve the robustness of the model, we selected the strongest 100 correlated features with brain age and employed them to train the Bayesian ridge model.

For better comparison and analyzing the effect of the regression model, we also evaluated popular regression models including: Kernel Ridge regressor^20^, ElasticNet^21^, Gradient Boosting regressor^22^, Support Vector Machine (SVM) regressor^23^, and Multi-layer Perceptron (MLP) regressor^24^.

#### Discovering important correlations

Last, to investigate the importance of each structure and feature for brain age estimation, we utilized the PFI technique^14^. The permutation feature importance is defined as the drop in a trained model score caused by randomly shuffled feature values. In other words, it tries to capture the importance of each feature by measuring the decrease in accuracy when one feature is randomly permuted with other features. Because this approach removes the link between the feature and the goal, the decline in the model score reflects how much the model is dependent on the feature. This technique reveals the most important structures and features for brain age estimation which can be used as a reliable landmark for brain age.

### Evaluation protocol

The accuracy of segmentation models was calculated using the average of Dice Coefficient Score (DSC)^40^ for all labels which measures the overlap between the generated segmentations and their corresponding ground-truth masks. For a given class, the DSC for a sample is formally defined as:

5 $$\begin{aligned} DSC = \frac{2|{\hat{\textbf{y}}} \cdot \textbf{y}|}{|{\hat{\textbf{y}}}|+|\textbf{y}|} \end{aligned}$$

where $\hat{\textbf{y}}$ is the discretized predicted segmentation, and $\textbf{y}$ is the ground-truth segmentation.

Two metrics were used to assess the performance of the brain age estimation models: coefficient of determination ($R^2$)^41^, which measures how well the predictions approximate the real ages, and Mean Absolute Error (MAE), which provides the average prediction error. For a set of *N* samples, these metrics are formulated as following:

6 $$\begin{aligned}{} & {} \textrm{MAE}=\frac{1}{N} \sum _{i=1}^{N}\left| z_{i}-\hat{z}_{i}\right| \end{aligned}$$

7 $$\begin{aligned}{} & {} \quad R^{2}=\left( \frac{N \sum _{i=1}^{N} z_{i}\hat{z}_{i}-\sum _{i=1}^{N} z_{i} \sum _{i=1}^{N} \hat{z}_{i}}{\sqrt{N \sum _{i=1}^{N} z_{i}^{2}-\left( \sum _{i=1}^{N} z_{i}\right) ^{2}} \sqrt{N \sum \hat{z}_{i}^{2}-\left( \sum _{i=1}^{N} \hat{z}_{i}\right) ^{2}}}\right) ^{2} \end{aligned}$$

### Compared methods

To evaluate the performance of the proposed method with respect to existing literature we included three recent relevant works in our empirical validation. These methods include: a model based on multiplanar slices and Test-Time Augmentation (2DTTA)^12^, an attention-based residual network (ARN)^11^ and a Global–Local Transformer (GLT)^10^. Note that these methods represent the state-of-the-art for image-based brain age estimation. Furthermore, it is noteworthy to mention that for all the methods, including 2DTTA, ARN, and GLT we run the experiments on the same data splits. Moreover, to have a fair comparison, we also searched for the best sets of hyperparameters and used the same data augmentation strategy for all tested methods.

### Implementation details

All segmentation networks and regression methods were implemented using PyTorch. We trained the segmentation network using Adam optimizer with a learning rate starting at $1\times 10^{-3}$, a weight decay of 0.5 every 20 epochs, and a batch-size of 32. Furthermore, these networks are trained on small 3D patches of sizes equal to $64\times 64\times 64$ voxels, following the standard literature in medical image segmentation. At test time, the final predicted segmentation is generated by sticking the segmentation of small patches together. Moreover, the regression models are based on the implemented models in scikit-learn library. Last, for all methods, we searched the optimal hyper-parameters on the independent validation set. Experiments were run in a server with 2 NVIDIA RTX A6000 GPU cards.

## Data Availability

DHCP dataset with all scans, preprocessed images and derivatives are available at http://www.developingconnectome.org/data-release/second-data-release/.

## Code availability

The implemented code for all steps will be available on GitHub upon acceptance of the paper.
